# Recent progress in functional modification and crosslinking of bioprosthetic heart valves

**DOI:** 10.1093/rb/rbad098

**Published:** 2023-11-06

**Authors:** Cheng Zheng, Li Yang, Yunbing Wang

**Affiliations:** National Engineering Research Center for Biomaterials, Sichuan University, Chengdu 610064, China; National Engineering Research Center for Biomaterials, Sichuan University, Chengdu 610064, China; National Engineering Research Center for Biomaterials, Sichuan University, Chengdu 610064, China

**Keywords:** bioprosthetic heart valves, modification, crosslinking, anticalcification, antithrombosis

## Abstract

Valvular heart disease (VHD), clinically manifested as stenosis and regurgitation of native heart valve, is one of the most prevalent cardiovascular diseases with high mortality. Heart valve replacement surgery has been recognized as golden standard for the treatment of VHD. Owing to the clinical application of transcatheter heart valve replacement technic and the excellent hemodynamic performance of bioprosthetic heart valves (BHVs), implantation of BHVs has been increasing over recent years and gradually became the preferred choice for the treatment of VHD. However, BHVs might fail within 10–15 years due to structural valvular degeneration (SVD), which was greatly associated with drawbacks of glutaraldehyde crosslinked BHVs, including cytotoxicity, calcification, component degradation, mechanical failure, thrombosis and immune response. To prolong the service life of BHVs, much effort has been devoted to overcoming the drawbacks of BHVs and reducing the risk of SVD. In this review, we summarized and analyzed the research and progress on: (i) modification strategies based on glutaraldehyde crosslinked BHVs and (ii) nonglutaraldehyde crosslinking strategies for BHVs.

## Graphical Abstract

**Figure:**
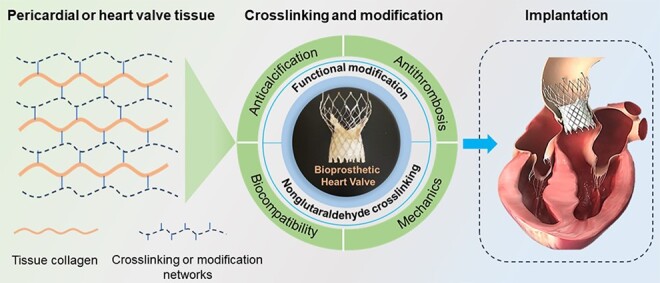

## Introduction

Valvular heart disease (VHD), with high morbidity and mortality in the elderly, is one of the most prevalent cardiovascular diseases. Patients with moderate to severe VHD account for nearly 2.5% of the global population [1]. The incidence of VHD increases with age: 0.7% in people aged 8–44 years and 13.3% in people aged 75 years and older [1]. The clinical manifestations of VHD were stenosis and regurgitation of native heart valve, which would lead to the abnormal physiological function of the heart, affect the normal life of patients. As there was no effective drug treatment, artificial heart valve replacement was recognized as current gold standard for the treatment of VHD [2–4].

With the coming of aging society, the number of patients with VHD will increase. Thus, the demand for artificial heart valve replacement surgery is increasing. Mechanical heart valves and bioprosthetic heart valves (BHVs) are two main categories of artificial heart valves clinically used to replace dysfunctional native heart valves of patients [5]. Mechanical heart valves are fabricated from synthetic materials with excellent mechanical strength and durability, while their hemodynamic performance is poor [6]. Mechanical heart valves are hydrodynamically deficient in two aspects: (i) the inner diameter of mechanical heart valves sometimes cannot match the natural valve root of patients with small aortic roots and (ii) the occluders of mechanical heart valves must be closed by reverse flow, which could cause regurgitant of mechanical heart valves [7]. Due to the complexity and risk of replacement surgery as well as thrombogenicity caused by poor hemodynamic performance of mechanical heart valves, mechanical heart valve replacement was no longer adaptable for patients with high risk of surgical thoracotomy and contraindications for lifelong anticoagulant therapy. BHVs have superior hydromechanical properties compared to mechanical heart valves, and patients do not need lifelong anticoagulation therapy after the implantation of BHVs. In addition, due to the rapid development of minimally invasive interventional therapy in recent years, transcatheter aortic valve replacement (TAVR) has been approved in clinics, further reducing the complexity and risk of artificial heart valve replacement. Despite higher total medical costs, TAVR was proved to be superior to surgical aortic valve replacement (SAVR) in terms of clinical outcomes and survival with comparable medical utilization [8]. The evolution of TAVR has led to widespread clinical implantation of BHVs.

Clinically used BHVs are mainly prepared from glutaraldehyde crosslinked xenobiological tissue (pericardium or aortic heart valve) [9, 10]. Although glutaraldehyde crosslinking improved the matrix stability, mechanical properties and durability of BHVs and reduced the immunogenicity of xenogeneic tissues, drawbacks associated with glutaraldehyde crosslinking, such as cytotoxicity, calcification, component degradation, mechanical failure and immune response would accelerate structural degradation and lead to structural valvular degeneration (SVD) of BHVs. These drawbacks would eventually shorten the service life of BHVs and raise the risk of secondary heart valve replacement surgery for patients [11–14]. Residual toxic aldehyde groups on glutaraldehyde crosslinked BHVs could devitalize cells and create calcification sites to induce calcification, initiate immune response and inhibit the endothelialization of BHVs after implantation. Owing to calcification, the function and lifespan of BHVs were seriously compromised [15]. In addition, the occurrence of thrombosis on BHVs could cause dysfunction, induce calcification and accelerate subsequent structural degeneration of BHVs [16, 17]. In recent years, much effort has been devoted to further improving the performance, reducing the risk of SVD. In this article, the progress in research of BHVs is reviewed from two main aspects: (i) modification strategies of BHVs that targeted to overcome various shortcomings caused by glutaraldehyde crosslinking (Table 1) and (ii) development of nonglutaraldehyde crosslinking strategies for BHVs to completely circumvent the negative effects and shortcomings of glutaraldehyde crosslinking (Table 2).

**Table 1.** Modification methods for BHVs

Modification method	Valve matrix	Modification agents	Targets			
			Hemocompatibility	Cytocompatibility	Anticalcification property	Mechanics
Blocking of residual aldehyde group	Bovine pericardium [19–21, 23–25, 28, 29], Porcine heart valve [22, 26, 27], Porcine pericardium [30, 31]	L-Glutamic acid [19, 24, 25], L-lysine [20, 22], L-arginine [21], L-glutathione [23], Urazole [24], Aminopropanehydroxy-diphosphonate [25], AOA [26, 27], Heparin [28], Sulfonated PEO [29], Aminated polymers [30], Dopamine-polyethylenimine polymer [31]	*In vitro* assay [19, 21, 23, 30, 31]	[23, 28–31]	Rat subdermal implantation [19–21, 23, 24, 28–31], Rat abdominal subcutaneous/supramuscular implantation [22], Rat ventral abdominal implantation [25, 26], Sheep valved grafts [27], Canine Vascular Model [29]	[21, 23, 24, 28–30]
Polysaccharide surface modification	Porcine pericardium [37, 41, 44, 45, 61], Porcine heart valve [39], Bovine pericardium [47, 48]	Chondroitin sulfate hydrogel [37], Heparin surface [39], Dopamine-modified alginate [41], VEGF-loaded hyaluronic acid hydrogel [44], Hyaluronic acid and chondroitin sulfate [45], Hyaluronic acid and polyacrylamide [61], Hyaluronic acid derivative [47, 48]	*In vitro* assay [37, 44, 45, 61]	[37, 44, 45, 61]	Rabbit subdermal implantation [39] Rat subdermal implantation [41, 44, 45, 61] Simulated body fluid immersion [47] Mice subdermal implantation [48]	[41, 47, 61]
Polymeric hydrogel network hybrid	Porcine pericardium [49, 50] Porcine heart valve [51, 52, 55, 56]	Poly-sodium acrylate hydrogel [49], Poly-2-methacryloyloxyethyl phosphorylcholine hydrogel [49], Poly-acryloyloxyethyltrimethyl ammonium chloride [49], Poly(ethylene glycol)methacrylate hydrogel [50], Poly(ethylene glycol) diacrylate hydrogel [51], Poly(ethylene glycol) [52], Poly(ethylene glycol) diacrylate and zwitterion (2-[methacryloyloxy]ethyl)dimethyl-(3-sulfopropyl) ammonium hydroxide [55], Poly-N-acryloyl glycinamide hydrogel [56]	*In vitro* assay [49, 50, 56]	[49, 50, 56]	Rat subdermal implantation [49, 50, 55, 56]	[49, 50, 52, 56]
Other anticalcification strategies	Porcine aortic heart valves [57–59, 62–64], Bovine Pericardium [60, 65, 66], Porcine pericardium [67]	Ethanol treatment [57, 58], Long chain alcohol [59], Organic solvents and amino acid [60], Ethanol and Aluminum Chloride [62, 63] Polyphenol and Ferric Chloride [67], Sodium bisulfite [64], Surfactants Tween 80/TritonX100 and sodium dodecyl sarsinate [65] Ethanol/ether/Tween 80 [66], N-dodecyl-β-D-maltoside/isopropanol [68]	*In vitro* assay [68]	[67, 68]	Rat subdermal implantation [57–59, 63, 64, 66–68] Goat right ventricular outflow tract [60], Sheep valved grafts [57, 62, 63, 65]	

**Table 2.** Nonglutaraldehyde crosslinking method for BHVs

Nonglutaraldehyde Crosslinking method	Valve matrix	Crosslinking agents	Targets			
			Hemocompatibility	Cytocompatibility	Anticalcification property	Mechanics
Natural products-based crosslinking	Porcine heart valve [71, 72, 74], Bovine pericardium [75, 80], Porcine pericardium [82]	Procyanidin [71, 72], Nordihydroguaiaretic acid [73], Quercetin [74], Curcumin [75], Genipin [80], Sodium lignosulfonate [82]	*In vitro* assay [75, 82]	Cell culture [71, 73–75, 82]	Rat subdermal implantation [72, 75, 82], Simulated body fluid assay [74], Rabbit intramuscular implantation [80]	[71–75, 82]
Polysaccharide derivatives crosslinking	Bovine pericardium [83, 84, 86], Porcine pericardium [85]	Azide alginate [82], Sodium alginate dialdehyde [84], Pectin dialdehyde [85], Epoxidized chitosan [86]	*In vitro* assay [84–86]	[84–86]	Rat subdermal implantation [83–86]	[86]
Hybrid crosslinking	Porcine heart valve [90], Porcine pericardium [76]	EDC/NHS/neomycin sulfate [90], EDC/NHS/curcumin [76]	*In vitro* assay [76]	[76]	Rat subdermal implantation [76, 90]	[76]
Epoxy compounds crosslinking	Porcine heart valve [95]	Triglycidylamine [95]		[95]	Rat subdermal implantation [95]	
Isocyanate compounds crosslinking	Bovine pericardium [96, 97]	Hexamethylene diisocyanate/polyethylene glycol [96, 97]	*In vitro* assay [97]	*In vitro* calcification assay [97], Rat subdermal implantation [96]		
Photo-crosslinking	Porcine pericardium [98, 99]	Riboflavin/ultraviolet light [98], Rose-bengal/visible light [99]		[98, 99]	Rat subdermal implantation [98, 99]	
Silane coupling agents crosslinking	Porcine pericardium [100]	3‐glycidyloxypropyl trimethoxysilane [100]	*In vitro* assay [100]	[100]	Rat subdermal implantation [100]	
Oxazolidines crosslinking	Porcine pericardium [101]	Bicyclic hydromethyl-oxazolidine [101]	*In vitro* assay [101]	[101]	Rat subdermal implantation [101]	
Catechol crosslinking	Porcine pericardium [102]	3,4-Dihydroxybenzaldehyde [102]	*In vitro* assay [102]	[102]	Rat subdermal implantation [102]	[102]
Double-bond crosslinking	Porcine pericardium [103–112], Porcine heart valve [113, 114]	MA [103, 113], MA/SBMA [104], MA/3-sulfopropyl methacrylate [105], MA/Methacrylated hyaluronic acid [114], GMA [106], GMA/2-(perfluorooctyl)ethyl methacrylate [107], GMA/rhCOLIII [108], ICM [109], ICM/PEGDA [110], ICM/2-hydroxyethylmethacrylate [111], ICM/2-methacryloyloxyethyl phosphorylcholine [112]	[104, 105, 107, 108, 110, 111, 114]	[103–106, 108–114]	Rat subdermal implantation [103–114]	[109–112]

## Modification strategies based on glutaraldehyde crosslinked BHVs

### Blocking the residual aldehyde groups on BHVs

Glutaraldehyde crosslinking effectively improved the stability and mechanical performance of BHVs to resist the shear stress and enzymatic degradation caused by cyclic blood flow and host immune rejection respectively. However, toxic residual aldehyde groups would be inevitably left during the crosslinking process. The presence of residual aldehydes on BHVs could cause shortcomings of severe cytotoxicity, calcification, poor biocompatibility and thrombosis. These drawbacks could be significantly restrained by eliminating or blocking the toxic residual aldehydes on BHVs. At present, aldehyde condensation (amino compounds), aldehyde reduction and reducing amination were the most widely reported strategies for the elimination of residual aldehydes on BHVs. The toxic residual aldehydes were readily reduced and converted into hydroxyl groups in the presence of reducing agent sodium borohydride, and the cytotoxicity and calcification degree of BHVs could be relived after the aldehyde reduction treatment [18]. Amine-containing compounds such as natural amino acids (glutamic acid, glycine, arginine and lysine) and amine-terminated oligo peptide were also reported to block residual aldehydes through Schiff base formation reaction between amine group and residual aldehydes to improve the biocompatibility and anticalcification property of BHVs [19–23]. Moreover, this modification method was facile and might confer BHVs with superior biological activity of natural amino acids. Recently, Wu *et al*. [23] developed a new modification strategy using L-glutathione (GSH) to cap the toxic and calcification-inducive aldehyde residues of glutaraldehyde crosslinked BHVs, which markedly decreased the calcification level by 80% and lowered the inflammatory response of BHVs. Furthermore, after GSH (8 mmol/l) incubation treatment, the residual aldehyde group content was nearly reduced to zero; thus the endothelialization potential and biocompatibility of BHVs were also significantly improved [23]. In addition to natural amino acids, uridazole and amino-propane hydroxydiphosphonate could also be used to block residual aldehydes to further eliminate the cytotoxicity and calcification of BHVs [24, 25]. The efficiency of introducing amino-propane hydroxydiphosphonate to eliminate the aldehyde group improved with the increase in pH and reaction time. A compound (α-aminoleic acid) with hydrophilic amino group and hydrophobic fatty chains could react with residual aldehydes effectively [26, 27]. On the one hand, toxic residual aldehyde groups were effectively eliminated; on the other hand, the presence of fatty chains on α-aminoleic acid (AOA) significantly inhibited the diffusion of calcium ions into collagenous matrix of BHVs [27]. AOA could bind residual aldehyde groups on BHVs more firmly and inhibit the initial calcium nucleation, thus preventing calcification in the long term. Thus, AOA was widely used in aldehyde blocking and anticalcification treatment of commercial BHVs. Lee *et al.* [28] developed heparinized BHVs by reducing amination between the amino group on heparin and residual aldehydes of glutaraldehyde crosslinked BHVs in the presence of reducing agent sodium borohydride, which effectively eliminated the problems of cytotoxicity and severe calcification caused by residual aldehydes. Moreover, heparinized BHVs were expected to be more hemocompatible due to heparin’s inherent anticoagulant activity, and the antithrombotic property of BHVs was also pending evaluation. A relatively hydrophilic and inert surface on BHV was constructed by grafting sulfonate-terminated polyethylene oxide using reductive amination (the sulfur content has increased by 15%), which further reduced the inflammatory response and calcification of BHVs [29]. Aminated hydrophilic polymers such as aminated poly-phosphoacylcholine brushes with cellular membrane mimic function were also exploited to block the aldehydes to curb calcification, inhibit thrombosis and lower the inflammatory reaction on BHVs [30]. Dopamine and its related polymeric products were also capable of capping the residual aldehydes [31]. The dopamine-modified BHVs could serve as a scaffold for functional modification based on layer-by-layer modification technic [31]. Wang *et al.* [31] devised a rivaroxaban loaded endothelium-like coating on dopamine-polyethylenimine polymer pretreated BHVs’ matrix to confer BHVs with thrombin-responsive antithrombotic and antiinflammatory capacity. The calcification of BHVs treated by this method was also effectively resisted in rat model [31].

### Polysaccharide surface modification

BHVs were collagen-based cardiovascular biomaterials. The exposure of mineralizable hole zones on collagenous matrix was prone to serve as a calcification site and induce the calcification of BHVs [32, 33]. In addition, the inevitable unwanted adsorption of plasma proteins might mediate thrombosis and immune responses [34–36]. Some anionic polysaccharides such as sodium alginate, chondroitin sulfate, heparin and hyaluronic acid with excellent biocompatibility and antithrombotic performance were widely applied in surface modification of BHVs to shield the collagenous matrix. By immobilizing these hydrophilic hemocompatible polysaccharides, the interactions between calcium ions and calcific zone on BHVs were blocked, and unwanted adsorption of plasma proteins and its related thrombosis were also effectively inhibited. Biodegradable methacrylated chondroitin sulfate hydrogel was introduced to methacrylated BHVs through radical polymerization to facilitate the endothelialization and thromboresistance of BHVs, which might further promote the long-term biosafety of heart valve implantation [37]. Heparin, a clinically anticoagulative polysaccharide drug that was generally grafted on blood-contacting biomaterials [38], was also covalently immobilized to inhibit the calcification and coagulation on BHVs’ matrix [39]. Alginate was readily chelated with calcium ions to form dynamically crosslinked biomedical hydrogels [40]. Adhesive alginate (dopamine modified alginate) was introduced on BHVs and served as a dynamic protective barrier to curb the deposition of calcium ions on the collagenous matrix and further inhibit the calcification of BHVs [41]. Hyaluronic acid was a kind of acidic natural glycosaminoglycan with good biocompatibility and low immunogenicity, which was widely applied in surface modification of biomaterials [42, 43]. Hyaluronic acid was grafted to BHVs under the effect of carbodiimide amide condensation reagent, ferric ions, sodium trimetaphosphate, 1,4-butanediol diglycidyl ether and polymeric crosslinking network to improve the endothelialization potential, biocompatibility and acticalcification property of BHVs [44–46]. Adipic hydrazide-modified hyaluronic acid was also grafted on the surface of BHVs to inhibit the deposition of calcium ions [47, 48]. By using sodium trimetaphosphate and 1, 4-butadiol diglycyl ether as crosslinking agents, hyaluronic acid and chondroitin sulfate hydrogels were introduced into the glutaraldehyde crosslinked BHVs through *in situ* crosslinking to further improve the anticalcification and antithrombotic properties of BHVs [44, 45]. Wang’s group constructed a double-network hydrogel modification strategy for BHVs based on hyaluronic acid and polyacrylamide, which improved the antithrombogenicity and endothelialization potential of BHVs [46]. The durability of double-network hydrogel armed BHV was also proved to meet ISO 5840 [46]. Through the introduction of polysaccharides, the biocompatibility, hemocompatibility and anticalcification property of BHVs could be significantly improved [46]. The long-term *in vivo* performance or efficiency of these strategies is pending evaluation and research.

### Polymeric hydrogel network hybrid strategies for BHVs

**Figure 1.**
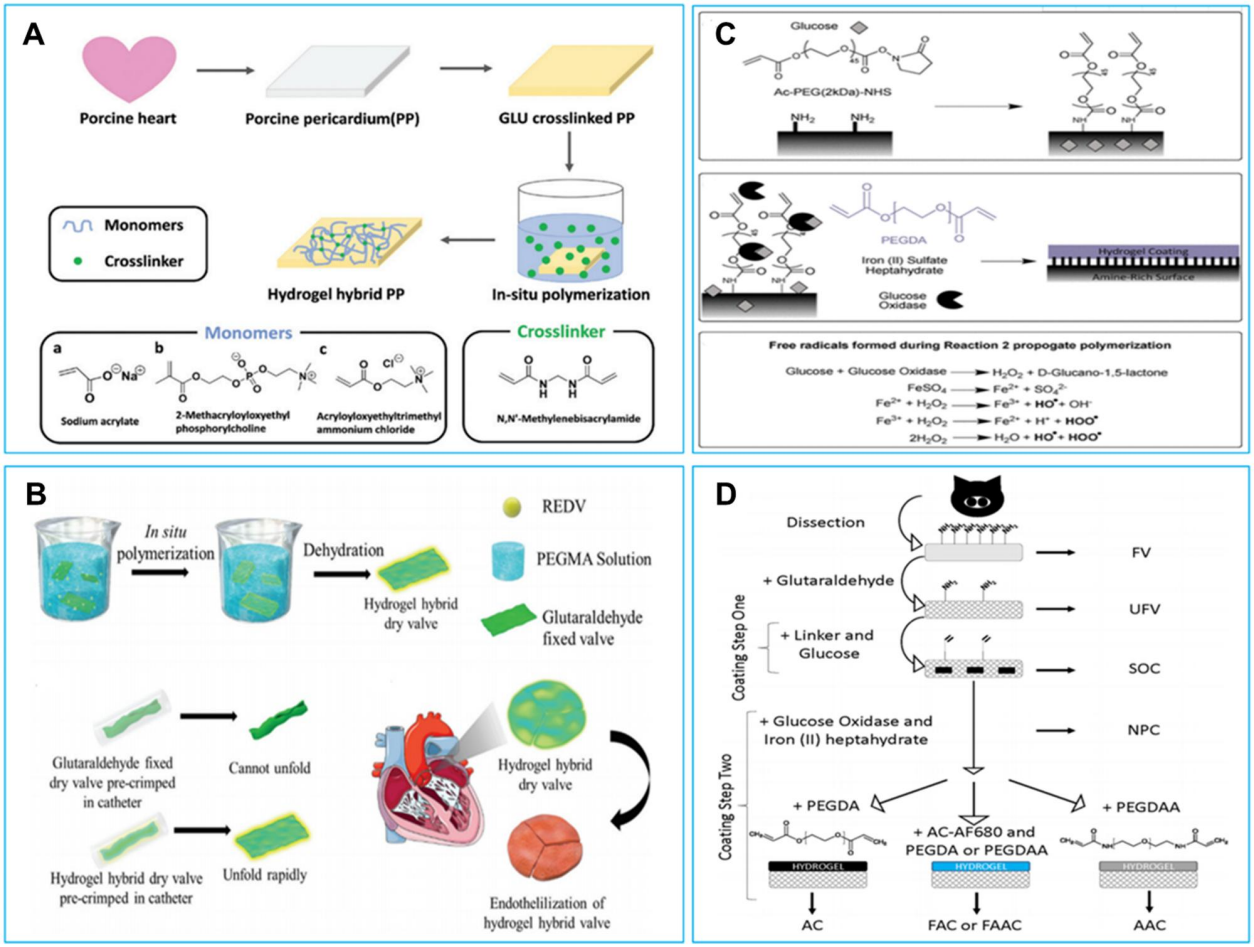
Strategies for modifying glutaraldehyde crosslinked bioprosthetic heart valves with polymer hydrogel networks. (**A**) Hydrogel hybrid BHVs by incorporation of polymers through *in situ* polymerization of monomers with different charges. Reproduced with permission from ref [49]. Copyright 2019 Royal Society of Chemistry. (**B**) PEGMA hydrogel hybrid pre-mounted valves. Reproduced with permission from ref [50]. Copyright 2020 Royal Society of Chemistry. (**C**) PEGDA interfacial coating hybrid bioprosthetic valve. Reproduced with permission from ref [51]. Copyright 2020 American Chemical Society. (**D**) PEG-based coatings for bioprosthetic valve tissues. Reproduced with permission from ref [52]. Copyright 2020 American Chemical Society.

The poor cytocompatibility and calcification of BHVs were also related to the way of preservation. Commercial BHVs were usually preserved in glutaraldehyde solution to resist microbial, while the invertible remaining glutaraldehyde was also toxic and might compromise the biocompatibility and accelerate the calcification of BHVs [53, 54]. A pre-mounted dry BHVs with the function of fast recovery under physiological conditions (hydrated state) would avoid the usage of glutaraldehyde preservation [50]. In recent years, polymer hydrogel network hybrid strategies were developed to improve the resilience, hemocompatibility, biocompatibility and anticalcification performance of glutaraldehyde crosslinked BHVs. Wang’s group prepared polymeric hydrogel hybrid BHVs (Figure 1A) through *in situ* polymerization of monomers with different charges (carboxylate anion, phosphocholine and quaternary ammonium cation monomer) to confer the BHVs with good resilience [49]. Furthermore, the prepared poly-phosphocholine hybrid BHVs exhibited better biocompatibility, antifouling properties as well as great potential for fabricating into a pre-mounted dry valve to avoid glutaraldehyde storage [49]. Through the *in situ* polymerization of poly(ethyleneglycol)methacrylate (PEGMA) on glutaraldehyde crosslinked BHVs, PEGMA polymer hybrid BHV was prepared (Figure 1B) [50]. The PEGMA polymer hybrid BHVs exhibited characteristics of low inflammatory response (secretion of pro-inflammatory factors decreased by ∼50%), low calcification (calcium content was reduced by ∼90%) and better resilience for pre-mounted dry valve [50]. Succinimide and acrylate terminated polyethylene glycol (NHS-PEG-Acrylate) was grafted on BHVs and polymerized with polyethylene glycol diacrylate (PEGDA) to obtain PEGylated hybrid BHVs. The PEGylated hybrid BHVs were reported to resist the adsorption of proteins (protein adsorption was reduced by ∼50%) (Figure 1C and D) [51, 52]. Polyethylene glycol diacrylate (PEGDA) and methacrylated sulphobetaine (SBMA) were hybrid with BHVs through *in situ* polymerization to resist the deposition of calcium and blood components [55]. N-acryloyl-glycinamide (NAGA) was also explored in the preparation of polymeric hydrogel (pNAGA) hybrid BHVs through *in situ* polymerization [56]. The pNAGA hybrid BHVs could be compressed into catheters in dry state and quickly recovered to their original shape under hydrated conditions without structural damage [56]. The introduction of pNAGA hydrogel also significantly improved the hydrophilicity of BHVs to resist thrombosis [56]. The incorporation of hydrogel networks effectively improved the biocompatibility, hemocompatibility and anticalcification properties of BHVs, while the binding stability between the polymeric hydrogel and BHVs, as well as anti-fatigue performance of these hybrid BHVs were pending further investigation.

### Other anticalcification strategies

The calcification degree of BHVs was also reduced by the usage of surfactants, inorganic salts and alcohol solution and other physical methods in the post-treatment of BHVs. Vyavahare *et al.* [57] reported that ethanol (80%, v/v) post-treatment could effectively inhibit the calcification of BHVs. Connolly *et al.* [58] reported that ethanol post-treatment could lower the calcification degree by reducing the calcification promoting components such as cholesterol and phospholipid on BHVs. Ethanol treatment might improve the anticalcification property of BHVs through three possible mechanisms: (i) ethanol treatment led to irreversible change of collagen configuration, thus reducing the calcification inducive sites; (ii) ethanol, as a good organic solvent, could extract molecules such as phospholipid and cholesterol which promote calcification from the valve tissue, thus reducing the calcification of BHVs’ matrix and (iii) ethanol could remove cell debris that generally serve as calcification site for BHVs [57]. Long aliphatic chain alcohol and ethanol could also effectively reduce the cholesterol content and calcification degree without affecting the mechanical properties of BHVs [59]. BHVs crosslinked in glutaraldehyde solution (with ethanol/octanol/water as solvent) also exhibited good anticalcification performance in large animal studies [60]. Aluminum ions (Al^3+^) were embedded on BHVs to further shield the calcific sites that could bind with calcium ions, thus inhibiting the deposition of calcium ions and calcification [63, 69]. Similarly, ferric ions (Fe^3+^) were also reported to lower the degree of calcification by occupying the calcium binding site on BHVs and inhibiting the deposition of calcium ions [67]. Sodium bisulfite solution treated BHVs were reported to exhibit improved anticalcification property, while the anticalcification mechanism of sodium bisulfite was not clear [64, 70]. Surfactants Tween 80, TritonX100 and sodium dodecyl sarsinate were synergistically used to treat BHVs, and the anticalcification effect was characterized and verified in sheep implantation model [65]. Additionally, combination usage of ethanol, ether and surfactant Tween 80 could effectively inhibit the calcification of BHVs by removing the cholesterol, free fatty acids and phospholipid and other calcification-promoting components from BHVs [66]. Recently, Ding’s group has put forward a novel biosurfactant-participated TSD (two-step decellularization) strategy to prepare BHVs with better anticalcification performance in which glutaraldehyde fixed BHVs’ material (bovine pericardium) was decellularized with mild biosurfactant n-dodecyl-β-D-maltoside and isopropanol [68]. The TSD treated BHVs’ materials exhibited relatively lower degree of calcification and better biocompatibility in rat subdermal implantation model, which might markedly reduce the risk of SVD [68].

## Nonglutaraldehyde crosslinking and modification strategies for BHVs

In addition to detoxification, anticalcification, anticoagulation and antiinflammatory modification strategies for BHVs based on glutaraldehyde crosslinking, nonglutaraldehyde crosslinking and modification strategies for BHVs were also widely explored to avoid the inherent cytotoxic residual aldehydes, calcification and inferior biocompatibility, thrombosis and other drawbacks of glutaraldehyde crosslinking. At present, research on non-glutaraldehyde crosslinking and modification for BHVs mainly include (Table 2): natural product-based crosslinking, polysaccharide derivative crosslinking, small molecule crosslinking (such as carbodiimide crosslinking agent, epoxy compound crosslinking agent, isocyanate, etc.), photocrosslinking, oxidative crosslinking of catechol, oxazolidines crosslinking and novel double-bond crosslinking methods. These nonglutaraldehyde crosslinking methods could avoid negative effects of residual aldehydes and free glutaraldehyde in conventional crosslinking process of BHVs. The nonglutaraldehyde crosslinked BHVs exhibited significantly improved cytocompatibility, hemocompatibility and anticalcification performance compared with traditional glutaraldehyde crosslinked BHVs.

### Natural products-based crosslinking strategies

**Figure 2.**
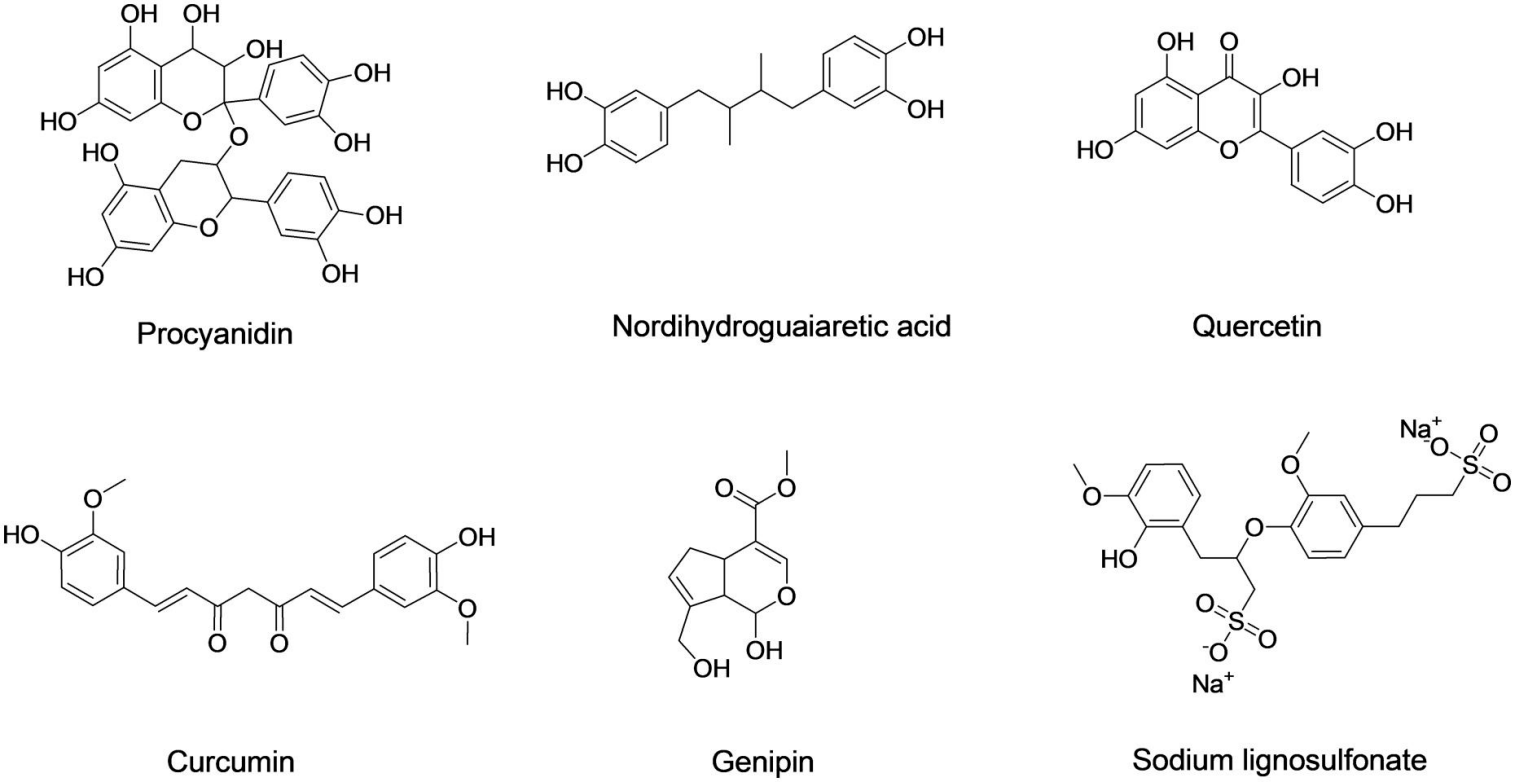
Chemical structures of natural product crosslinking agents for bioprosthetic heart valves.

In recent years, a series of natural products have been reported as new crosslinking agents for BHVs (Figure 2), providing new ideas for the development of nonglutaraldehyde crosslinking strategies. Procyanidin could stabilize the components of BHVs to a certain extent, and procyanidin crosslinked BHVs showed excellent anticalcification performance in rat subdermal implantation model [71, 72]. However, rapid release of procyanidin from crosslinked matrix might lead to further compromise of their crosslinking degree and component stability. Nordihydroguaiaretic acid could effectively stabilize the collagen matrix and enhance the tensile strength of BHVs [73]. In addition, nordihydroguaiaretic acid crosslinked BHVs exhibited better endothelial cell compatibility, which was conducive to fast endothelialization and improvement of biocompatibility and hemocompatibility [73]. Though nordihydroguaiaretic acid crosslinked BHVs exhibited lower risk of degeneration, their long-term stability and anticalcification properties were not characterized. Flavonol compound quercetin was also exploited to stabilize the component and improve the thermal stability of BHVs [74]. The mechanical performance, anticalcification property and cytocompatibility of BHVs obtained by quercetin crosslinking were better than those of BHVs crosslinked by glutaraldehyde [74]. The hemocompatibility of nordihydroguaiaretic acid or flavonol compound crosslinked BHVs needs to be evaluated to verify their clinical potential. Curcumin, as a flavonoid small-molecule drug with a variety of pharmaceutical activities, has also been used as a crosslinking agent for BHVs in recent years [75–77]. The curcumin crosslinked BHV showed excellent anticalcification performance, endothelialization potential and anti-adhesion properties against platelets [75, 76]. While the poor component stability of curcumin crosslinked tissue against collagenase degradation needed to be concerned before further application [75, 76]. Genipin is a natural product of iridoid glycosides extracted from gardenia, which can react with amino-containing biological macromolecules (such as chitosan, enzymes and collagen) and serve as a natural crosslinking agent for them [78, 79]. When genipin was used to crosslink BHVs, it was found that genipin crosslinking significantly enhanced the maximum fracture tensile strength and reduced the calcification and inflammation of BHVs [80, 81]. However, the cytocompatibility, hemocompatibility, long-term stability and safety of genipin crosslinked BHVs need to be evaluated to verify their application potential. Recently, sodium lignosulfonate was also exploited to crosslink and modify BHVs [82]. Zhang *et al.* [82] found that sodium lignosulfonate crosslinked BHVs had excellent cytocompatibility, antithrombotic and anticalcification properties, which further enriched the research of natural crosslinking agents for BHVs. The crosslinking mechanism of sodium lignosulfonate crosslinking was not well-demonstrated, thus the long-term components stability of sodium lignosulfonate crosslinked BHVs needed to be further evaluated and enhanced.

### Polysaccharide derivatives crosslinking strategies

Polysaccharide derivatives containing functional groups that are reactive towards amino groups on BHVs’ matrix could be applied in crosslinking of BHVs to further improve the biocompatibility, hemocompatibility and anticalcification performance of BHVs. Polysaccharides derivatives with crosslinking properties were obtained through the grafting or conversion of azide, aldehyde and epoxy groups. Azide-grafted sodium alginate (azide-alginate) was prepared and then reacted with amino groups of BHVs to achieve crosslinking [83]. The *in vivo* calcification degree of azide-alginate crosslinked BHVs was significantly lower than that of glutaraldehyde crosslinked BHVs [83]. Sodium alginate dialdehyde was prepared using oxidative sodium periodate and applied in crosslinking of BHVs [84]. Sodium alginate dialdehyde crosslinking could stabilize the collagen matrix on BHVs, inhibit platelet adhesion and improve the anticalcification performance and cytocompatibility of BHVs [84]. Similarly, pectin was also oxidized into pectin dialdehyde and explored as an alternative for glutaraldehyde in the crosslinking of BHVs [85]. Pectin dialdehyde crosslinking has markedly improved the cytocompatibility and lowered the calcification degree of BHVs in rat subdermal model [85]. Epoxidized chitosan was also prepared and used as crosslinking agent for BHVs [86]. The epoxidized chitosan crosslinked BHV exhibited better cytocompatibility and could inhibit the calcification *in vivo* [86]. Owing to reversible bond (Schiff base) and biodegradability of polysaccharides crosslinking network, the long-term stability of crosslinked BHVs was also needed to be concerned. Polysaccharide derivatives crosslinking strategies have exploited the hydrophilic and biocompatible backbone of polysaccharides as crosslinking network, which significantly improved the hemocompatibility and biocompatibility of BHVs.

### Hybrid crosslinking technology

Carbodiimides can react with carboxyl groups, while the O-acylurea intermediates are highly reactive and prone to hydrolysis. To stabilize the O-acylurea intermediates and facilitate the condensation reaction with amine groups, N-hydroxysuccinimide (NHS) was added to react with O-acylurea derivatives and form a relatively stable succinimide ester intermediates. Based on the high reactivity of carbodiimide to carboxyl groups on collagen-based BHVs, carbodiimide derivative hydrochloride 1-(3-dimethylaminopropyl)-3-ethylcarbodiimide hydrochloride (EDC) was commonly used in combination with NHS to crosslink BHVs [87, 88]. Luo *et al.* [88] treated BHVs’ matrix with sodium lauroylsarcosinate and EDC/NHS to stabilize the BHV’s components and improve BHVs’ anticalcification property. The sodium lauroylsarcosinate was exploited as decellularization agent for BHVs [88]. Leong *et al.* [89] crosslinked BHV with EDC/NHS and neomycin sulfate, which could improve their cytocompatibility, anticalcification properties and further reduce the risk of structural degeneration caused by glycosaminoglycan degradation. Yang *et al.* [76] reported that BHVs simultaneously crosslinked by curcumin and EDC/NHS exhibited significantly improved stability and anticalcification property in rat model. Moreover, this BHV could quickly rehydrated and rebounded after being folded in dry state, suggesting that it exhibited good resilience and had great potential to be fabricated into pre-mounted dry BHVs [76]. Tam *et al.* [90, 91] developed a triple crosslinked method (TRI) using EDC/NHS, neomycin sulfate and 1,2,3,4,6-penta-o-galloyl-beta-d-glucopyranose. The BHVs crosslinked by TRI method had higher stability against multiple enzymatic (collagenase, elastase and glycosaminosanase) degradation and exhibited excellent tear resistance after enzyme degradation treatment [90, 91]. Moreover, the biocompatibility and anticalcification properties of TRI crosslinked BHVs were better than those of glutaraldehyde crosslinked BHVs. The EDC/NHS crosslinked BHVs exhibited good biocompatibility and low calcification degree, which lowered the risk of SVD caused by calcification.

### Epoxy compounds crosslinking strategies

Different from aldehyde compounds, epoxide can react with amino, hydroxyl, carboxyl groups on collagen to form more stable (C–N or C–O) bonds and avoid toxic aldehyde residues. Epoxide was more readily to undergo ring-opening reaction due to high stain of three membered ring in terms of chemistry [92]. Under acidic conditions, epoxide was mainly attacked by nucleophilic carboxyl groups on the collagen matrix [93]. In contrast, amine groups were converted or consumed by ring-opening of epoxides under basic conditions [93]. Relatively basic conditions (pH = 8) and longer reaction time (more than 48 h) were favored for achieving improved crosslinking degree [94].

Thus, epoxide compounds were widely studied in the crosslinking of BHVs to serve as a substitute for glutaraldehyde. Multifunctional epoxide compounds could effectively improve the component and thermal stability of BHVs, and the crosslinked BHVs exhibited low calcification degree [115–118]. Connolly *et al.* [95] synthesized a trifunctional epoxyl crosslinking agent triglycidylamine and applied it to crosslink BHVs. The component stability (*in vitro* resistance to enzymatic degradation) of triglycidylamine crosslinked BHVs was equivalent to that of glutaraldehyde crosslinked one [95]. Moreover, the biocompatibility and anticalcification properties of triglycidylamine crosslinked BHVs were better than those of glutaraldehyde crosslinked BHVs [95]. Mercapto-bisphosphonate was an anticalcification bisphosphonate [119]. Thiol groups on thiolated bisphosphonate were also used to cap the residual epoxy groups on triglycidylamine crosslinked BHV [120]. They achieved the removal of unwanted epoxy group residues and the introduction of anticalcification bisphosphonate fragments simultaneously, which further reduced the calcification degree and cytotoxicity of BHVs [120]. The *in vitro* stability of epoxide compounds crosslinked BHVs was comparable to that of glutaraldehyde crosslinked BHVs, and their biocompatibility and anticalcification performance were superior to those of glutaraldehyde crosslinked BHVs [58]. However, the structural instability *in vivo* has limited the application of epoxide compounds crosslinked BHVs as substitutes for glutaraldehyde crosslinked BHVs [58]. The *in vivo* stability of BHVs crosslinked by epoxide compounds still needs to be further improved for further application.

### Isocyanate compounds crosslinking

Isocyanate group is a kind of functional group with strong electrophilicity, which can produce stable urea bonds and carbamate bonds by condensation reaction with nucleophilic groups such as amino group and hydroxyl group. Hexamethylene diisocyanate (HDI) contains two isocyanate groups, which can be applied in crosslinking of amine-rich biomacromolecules such as chitosan and collagen [121, 122]. HDI, with similar aliphatic backbone to glutaraldehyde, is a bifunctional crosslinking agent with high reactivity and can be applied in crosslinking of BHVs. Vasudev *et al.* [97] reported that HDI crosslinked BHVs exhibited low calcification degree. By further grafting polyethylene glycol on HDI crosslinked BHVs, the calcification degree of BHVs could be further reduced to a relatively lower level [97]. Moreover, it was found that glutaraldehyde crosslinked BHVs also exhibited anticalcification properties after secondary crosslinking by HDI [96]. However, isocyanate groups were readily hydrolyzed in aqueous solution, which limited the research and application of isocyanate compounds as crosslinking agents for BHVs. By regulating the solubility of isocyanate crosslinking agents in the crosslinking system of BHVs and reducing the side reactions between crosslinking agents and solvents, the research on isocyanate compounds crosslinking agents for BHVs might be further promoted.

### Photo-crosslinking strategies

**Figure 3.**
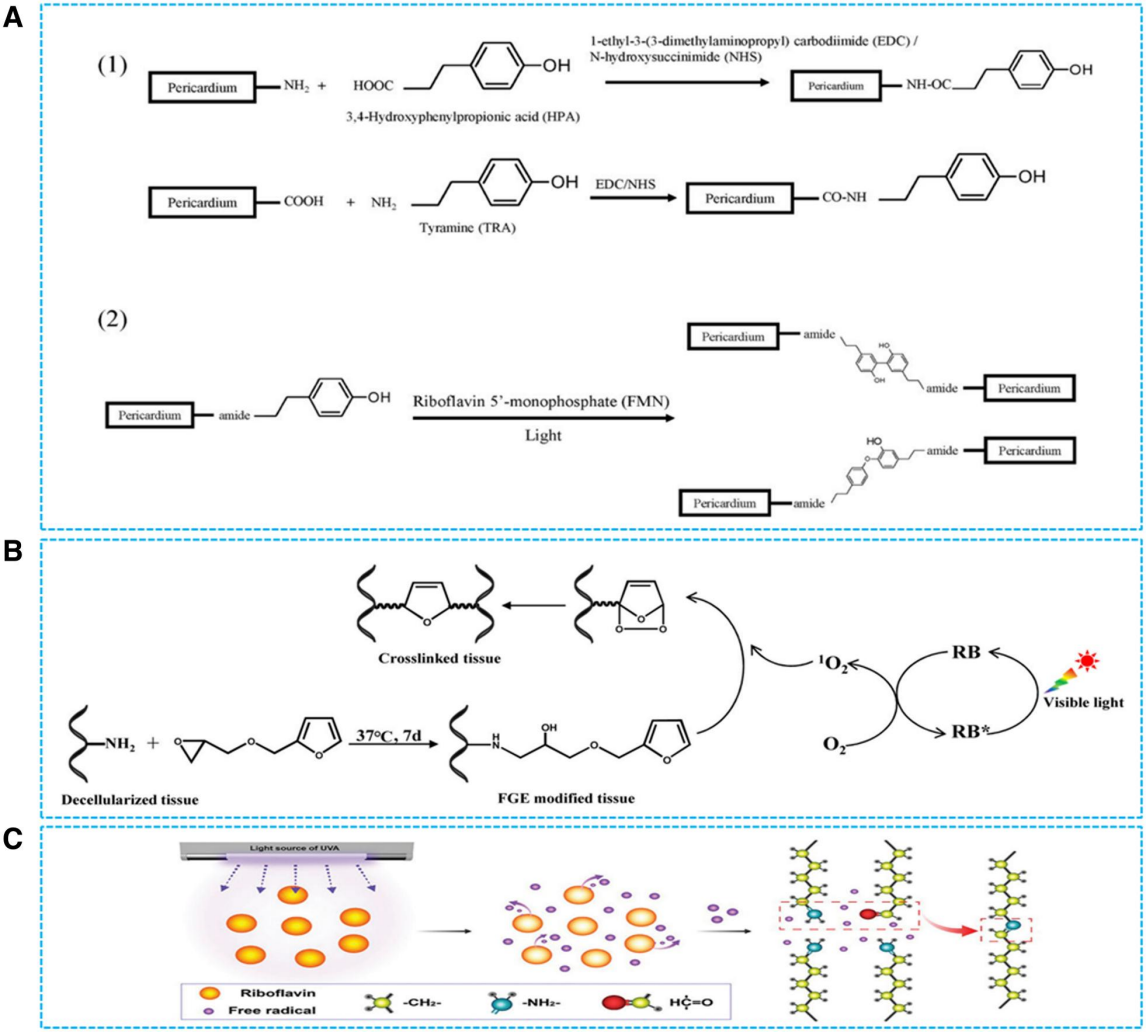
Photo-crosslinking strategies for bioprosthetic heart valves. (**A**) Riboflavin photo-crosslinking method for phenol grafted BHV matrix. Reproduced with permission from ref [98]. Copyright 2019 John Wiley and Sons. (**B**) Visible light-induced crosslinking of BHVs. Reproduced with permission from ref [99]. Copyright 2022 John Wiley and Sons. (**C**) Ultraviolet-induced crosslinking of BHVs. Reproduced with permission from ref [123]. Copyright 2020 Royal Society of Chemistry.

Biomacromolecules modified with tyramine and its analogs could undergo cross-coupling reactions between the grated phenolic groups through riboflavin-mediated photo-crosslinking [124, 125]. Lei *et al.* [98] reported that coupling reactions (Figure 3A) between phenol groups on tyrosine and p-hydroxyphenylpropionic acid grafted BHVs were initiated under the catalysis of riboflavin and ultraviolet light to achieve the crosslinking of BHVs. This crosslinking strategy for BHVs allowed for greater utilization of reactive groups (amine and carboxyl groups) on BHVs compared with the conventional glutaraldehyde crosslinking method [98]. Elastin on BHVs’ matrix was effectively stabilized to avoid the elastin degradation-induced calcification [98]. However, the low mechanical strength of crosslinked BHVs suggested low crosslinking efficiency under the catalysis of riboflavin and ultraviolet light [98]. Yang *et al.* [99] developed a crosslinking method for BHVs (Figure 3B) based on rose-bengal-mediated photooxidation of furan. They exploited epoxy group-containing furan derivatives as crosslinking agents to covalently introduce furan ring [99]. Crosslinking of BHVs was achieved through visible light irradiation of furan grafted matrix [99]. The BHVs prepared by rose-bengal-mediated furan photooxidative crosslinking method exhibited higher component stability and good mechanical properties such as tensile strength and elongation, which indicated its superior crosslinking efficiency under the catalysis of rose-bengal [99]. Furthermore, the crosslinked BHVs exhibited lower calcification levels and significantly improved cytocompatibility and endothelialization potential [99]. Crosslinking of BHVs could also be achieved through ultraviolet (UV) irradiation of riboflavin-pretreated BHVs’ matrix (Figure 3C) [123]. The riboflavin-UV crosslinked BHVs had improved biocompatibility, which could facilitate the adhesion and growth of endothelial cell [123]. These photo-crosslinking methods exhibited the advantages of low cytotoxicity and anticalcification, which might potentially prolong the lifespan of BHVs.

### Silane coupling agents crosslinking

3‐glycidyloxypropyl trimethoxysilane (GPTMS) is a novel inorganic crosslinking agent that possesses epoxy group and trimethoxysilane groups. The epoxy group on GPTMS can undergo ring-opening reactions with nucleophilic groups (such as amino and hydroxyl groups) on the matrix of BHVs, and the grafted trimethoxysilane groups can form a silicon–oxygen–silicon crosslinking network structure. It was commonly used for crosslinking natural biopolymer-based materials such as chitosan, collagen and gelatin to prepare organic–inorganic hydrogel biomedical materials. Based on this, GPTMS was applied in crosslinking of BHVs [100]. The GPTMS crosslinked BHVs exhibited high collagen stability, low cytotoxicity, low degree of calcification and improved endothelialization potential, which were expected to extend the lifespan of BHVs [100]. The hydrolysis and other side reactions of epoxy and trimethoxysilane groups during crosslinking process should also be concerned and controlled. In addition, the long-term anticalcification performance, hydrodynamics and durability were also pending evaluation.

### Oxazolidines crosslinking

Oxazolidine and its derivatives have a methylene carbon atom that is simultaneously connected to a heteroatom nitrogen (secondary or tertiary amine nitrogen) and an oxygen bond in their structure [126]. The methylene carbon has strong electrophilicity and can react with amino groups on collagen, thereby crosslinking the collagen-based biomaterials [126]. Based on the crosslinking effect of oxazolidine on collagen-based materials, Yu *et al.* [101] synthesized bicyclic hydromethyl-oxazolidine through a one-step dehydration condensation process using trimethylolaminomethane and polyformaldehyde as raw materials. The bicyclic hydromethyl-oxazolidine was then exploited to crosslink BHVs [101]. The BHVs crosslinked by bicyclic hydromethyl-oxazolidine had comparable stability and mechanical properties to those of glutaraldehyde crosslinked BHVs and exhibited excellent biocompatibility, hemocompatibility and anti-calcification properties [101]. Meanwhile, the bicyclic hydromethyl-oxazolidine crosslinked BHV exhibited appropriate hemodynamic performance and durability, making it a potential substitute for glutaraldehyde-crosslinked BHV [101]. In addition, bicyclic ethyl oxazolidine was also reported to exhibit similar crosslinking effects on BHVs as bicyclic hydromethyl-oxazolidine [127]. Given the high reactivity of oxazolidine and its derivatives with collagen, more oxazolidine derivatives were expected to be ulteriorly studied as potential nonglutaraldehyde crosslinking agents for BHVs, and more facile synthesis methods for oxazolidine derivatives were also needed to be developed. For further application of oxazolidines crosslinking strategies, long-term animal studies are still needed to evaluate the safety, efficiency as well as bio- and hemocompatibility of the BHVs fabricated from oxazolidine derivatives crosslinked tissue matrix.

### Catechol crosslinking

Catechol groups are more readily to undergo coupling reactions under oxidative conditions, which are widely applied in coupling or crosslinking of biomacromolecules. Based on the principle of oxidative-coupling reaction of catechols and high reactivity between aldehydes and amine groups, Wu *et al.* [102] explored 3,4-dihydroxybenzaldehyde (DHBA) as a crosslinking agent to achieve the crosslinking of BHVs. In the first step, the aldehyde group of DHBA reacted with amino groups to covalently introduce a catechols on BHVs (pericardial). In the second step, grafted catechols were coupled under the initiation of oxidative agents to achieve the crosslinking process, resulting in DHBA crosslinked BHVs. Compared with glutaraldehyde crosslinked BHVs, DHBA crosslinked BHVs showed better cytocompatibility, hemocompatibility, and anticalcification performance, and their hydrodynamic performance was also proved to meet the requirements of ISO 5840 [102]. The adhesion of proteins and platelets was significantly inhibited in DHBA crosslinked BHV, resulting in improved antithrombogenicity [102]. However, the collagen stability of DHBA crosslinked porcine pericardium was slightly inferior to that of glutaraldehyde crosslinked porcine pericardium, which might be attributed to a lower amino conversion rate in the crosslinking process and relatively lower crosslinking efficiency. For advanced application in future studies, the durability of DHBA crosslinked BHVs should be tested, and the crosslinking efficiency of this method should also be further improved. Therefore, the crosslinking strategy based on catechol oxidation coupling still needs to be further improved and optimized. Converting the aldehyde group in DHBA into a more reactive functional group towards amino groups might be beneficial to further improve the efficiency of catechol crosslinking strategy.

### Double-bond crosslinking strategy for BHVs

**Figure 4.**
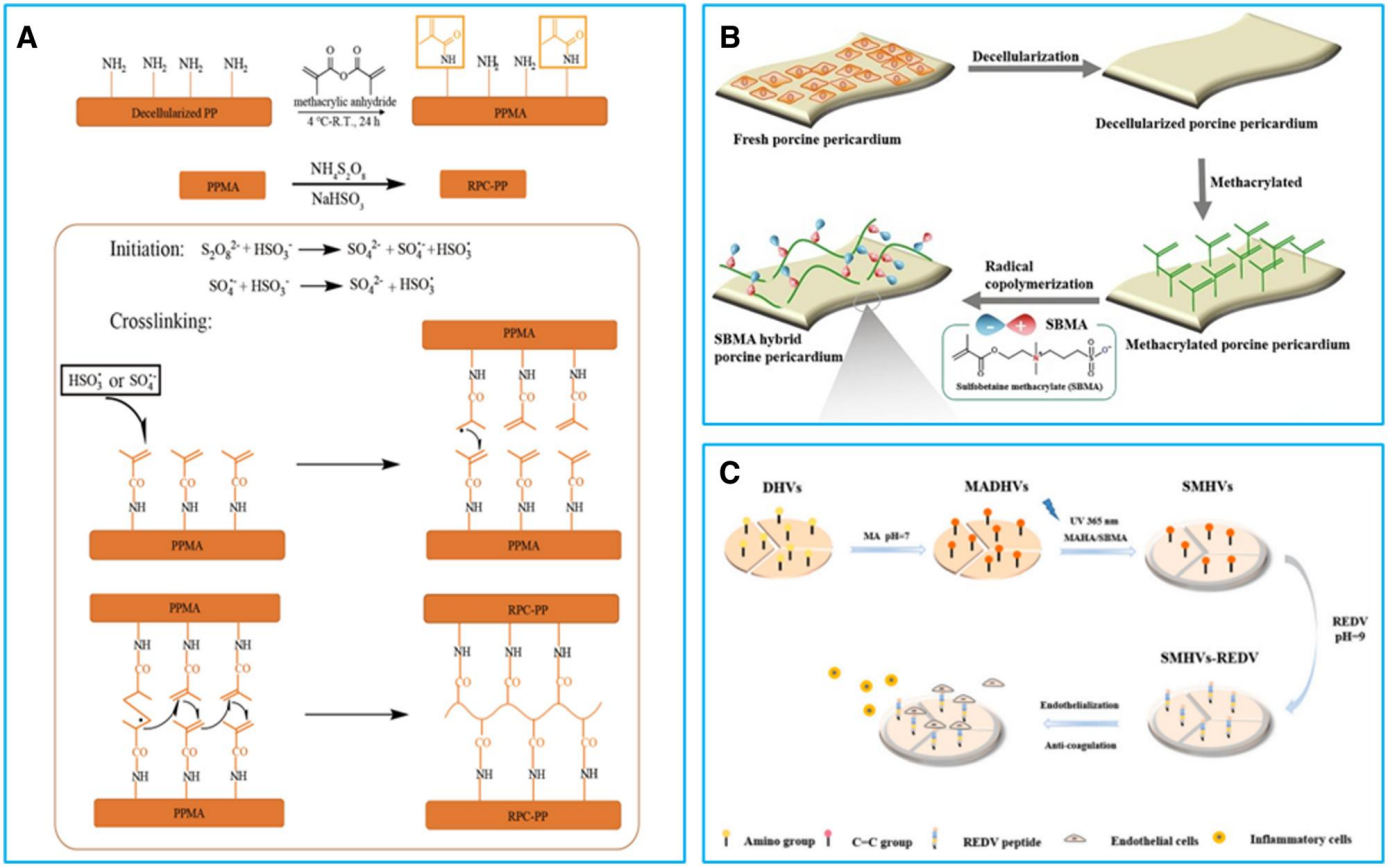
Double-bond crosslinking strategies for bioprosthetic heart valves based on methacrylic anhydride. (**A**) MA-based double-bond crosslinking strategy. Reproduced with permission from ref [103]. Copyright 2018 Elsevier. (**B**) Preparation of poly-SBMA hybrid BHV. Reproduced with permission from ref [104]. Copyright 2021 Elsevier. (**C**) Preparation of REDV-loaded zwitterionic hydrogel functionalized BHV. Reproduced with permission from ref [114]. Copyright 2021 Elsevier.

**Figure 5.**
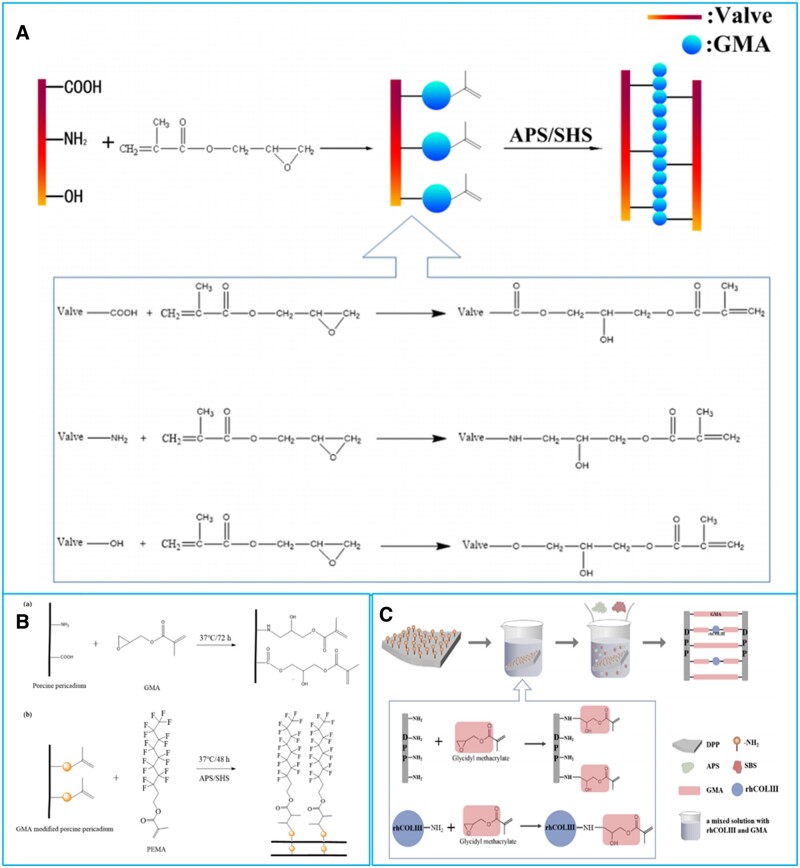
Double-bond crosslinking strategies for BHVs based on glycidyl methacrylate. (**A**) GMA-based double-bond crosslinking method. Reproduced with permission from ref [106]. Copyright 2019 American Chemical Society. (**B**) Preparation of hydrophobic polymer hybrid BHV. Reproduced with permission from ref [107]. Copyright 2021 John Wiley and Sons. (**C**) A rchcol III composited BHV obtained by GMA-based double-bond crosslinking. Reproduced with permission from ref [108]. Copyright 2022 Elsevier.

Due to its high efficiency and stable crosslinking structure, radical polymerization based on C = C (double bond) has been widely used in the field of novel nonglutaraldehyde crosslinking strategies for BHVs in recent years. Wang’s group has developed a series of double-bond crosslinking strategies for BHVs. In the double-bond crosslinking, BHVs’ matrix was firstly modified with double bond (methacrylate and acrylate) and then treated with initiator solution to achieve the crosslinking and functionalization of BHVs [128]. With the introduction of functional monomer in polymerization step, functionalization and crosslinking could be achieved simultaneously [128]. Wang’s lab first reported the double-bond crosslinking strategy (Figure 4A) based on methacrylic anhydride (MA) to stabilize the BHVs’ matrix [103, 113]. The MA crosslinked BHVs showed enhanced resistance to enzymatic degradation, biocompatibility and anticalcification properties over glutaraldehyde crosslinked BHVs [103, 113]. Based on the radical polymerization of methacrylate groups, zwitterionic monomer methacrylated sulfobetaine (SBMA) was applied in the polymerization process of methacrylated BHVs’ matrix to obtain a poly-SBMA hybrid BHV (Figure 4B) [104]. The poly-SBMA hybrid BHV was shown to resist calcification and thrombosis effectively, and its durability and hydrodynamics were ulteriorly proved to meet ISO 5840 in accelerated fatigue and pulsatile flow tests [104]. Ma’s group devised REDV-loaded zwitterionic hydrogel functionalized BHV (Figure 4C) through photo-induced copolymerization of methacrylated hyaluronic acid, SBMA and methacrylated porcine heart valve to improve the antithrombogenicity, anticaldification property and promote endothelialization [114]. Similarly, heparin-mimic monomer 3-sulfopropyl methacrylate potassium (SPM) was copolymerized with methacrylated porcine pericardium to prepare a poly-SPM hybrid nonglutaraldehyde BHV [105]. The introduction of poly-SPM markedly improved hydrophilicity of BHVs and further resisted the adhesion of plasma proteins and platelets, thereby enhancing the antithrombotic performance of BHVs [105]. Additionally, the inflammation reaction and calcification on poly-SPM hybrid BHV were significantly lower in rat model [105]. The hydrodynamic performance of poly-SPM hybrid BHV was proved to meet the requirements of ISO 5840 for BHVs under simulated conditions [105]. Glycidyl methacrylate (GMA), with epoxy group and methacrylate, was also exploited to covalently immobilize methacrylate on BHVs’ matrix in the double-bond crosslinking strategy (Figure 5A) [106]. BHVs prepared by the GMA-based double-bond crosslinking method were more stable and biocompatible than glutaraldehyde crosslinked BHVs [106]. Hydrophobic polymer hybrid BHV (Figure 5B) was prepared by *in situ* polymerization of GMA-modified porcine pericardium and hydrophobic fluorine-containing monomer [107]. The introduction of hydrophobic polymer conferred BHV with excellent antifouling and anticalicification property [107]. Through *in situ* polymerization of GMA-modified porcine pericardium and GMA-modified recombinant human type III collagen (rchcol III), a rchcol III composited BHV with enhanced hemocompatibility and biocompatibility was also obtained (Figure 5C) [108].

Based on the relatively high reactivity of isocyanate towards amine and hydroxyl groups on BHVs’ matrix, 2-isocyanatoethylmethacrylate (ICM), an agent with isocyanate and methacrylate (double bond) group, was also applied in double-bond crosslinking strategy for BHVs [109]. The ICM crosslinked BHV exhibited enhanced collagen stability, mechanical strength, biocompatibility and better anticalcification property [109]. Furthermore, the hydrodynamic performance and durability of ICM crosslinked BHV were satisfied with ISO 5840, which indicated the efficiency and application potential of ICM-based double-bond crosslinking strategy for BHVs [109]. To confer the BHVs with antithrombotic property, hydrophilic polymers were grafted on BHVs through copolymerization of monomers (hydroxyethyl methacrylate and poly(ethylene glycol) dimethacrylate) and ICM-modified pericardial matrix to resist the adsorption of blood components [110, 111]. With the introduction of polymers, the component stability of BHVs against enzymatic degradation was markedly enhanced [110, 111]. Cytomembrane biomimetic phosphorylcholine moiety was also introduced on BHV through copolymerization of 2-methacryloyloxyethyl phosphorylcholine and ICM-modified matrix [112]. The obtained phosphocholinated BHV was found to exhibit better antithrombogenicity, durability and endothelialization potential, which exhibited good application prospect [112]. Since BHVs are long-term cardiac implants, long-term hemocompatibility and anticalcification properties of double-bond crosslinked BHVs are also needed to be concerned. As there are more crosslinking and functional modification methods developed based on double-bond crosslinking strategy, the long-term *in vivo* performance of the obtained BHVs is pending systematic evaluation for further human implantation and clinic application.

## Challenges and future perspective

As the population ages, the number of patients with VHD is on the rise worldwide, and the demand for artificial heart valve replacement is also increasing. BHVs are pyramidally implanted in clinics due to their superior fluid dynamic performance as well as the convenience and safety of transcatheter aortic heart valve replacement. The increase in the average life expectancy brings higher requirements for the service life of BHVs. Despite that much effort has been devoted to reducing the risk of calcific SVD, the durability and anti-fatigue performance of BHVs are also pending further enhancement to meet the higher requirement of service life. The SVD of BHVs was also greatly associated with a series of immune reactions between patients’ immune systems and BHVs, the immune response initiated by BHVs should not be ignored. To further expand the implantation of BHVs in the younger patient population whose immune system is more vigorous than that of the elderly, the immune response-mediated SVD is urgently needed to be reduced or circumvented. In addition, the thorough removal of the immunogenic substances and factors on the current xenogeneic matrix of BHVs and the preparation of an immunogenicity-free matrix for BHVs are another challenge for the fabrication of BHVs [129].

Though there are more modifications and glutaraldehyde-free crosslinking strategies which could confer BHVs with good biocompatibility, anticalcification property, antithrombogenicity and appropriate durability, long-term animal experimental assessments are still needed to further evaluate their efficiencies and advantages before replacing glutaraldehyde crosslinking. A healthy endothelium formed on implanted BHVs might serve as a long-term natural physiological barrier to block the interactions between BHVs’ matrix and host, resulting in improved hemocompatibility and biocompatibility. How to achieve the fast endothelialization of BHVs under physiological conditions is another major challenge. Furthermore, the correlations between BHV’s durability and different crosslinking and modification methods also needed to be better clarified, which might promote the fabrication of more durable BHVs. Besides this, autologous valve tissues regenerated following the implantation of BHVs with regenerative properties might effectively inhibit thrombosis and immune response. Hence, developing a new generation of BHVs that exhibit the function of regeneration is a new direction for the research of BHVs through functional modification and crosslinking.

## Conclusions

With the development of transcatheter heart valve replacement, BHVs, with superior hemodynamic performance and lower thrombogenicity, are widely implanted in clinic. However, the drawbacks of glutaraldehyde crosslinked BHVs, including cytotoxicity, calcification, immune response, components degradation and thrombosis, might accelerate the SVD and shorten the lifespan of BHVs. We have summarized and reviewed the modification and crosslinking strategies that sought to reduce or circumvent the risk of SVD. The reported functional modification strategies and nonglutaraldehyde crosslinking strategies were mainly focused on the improvement of cytocompatibility, antithrombogenicity and anticalcification property of BHVs. Immune response is another important factor that facilitated the SVD of BHVs, while rare research works concerned the immunogenicity of BHVs and the elicited immune response. As BHVs were long-term cardiac implants that were expected to maintain their normal function for a relatively long time, improved durability as well as long-term anticalcification and antithrombotic properties were absolutely necessary. Thus, additional attention and investigations should be focused on suppression of immune response, rapid endothelization prolongation of durability and regenerative property of BHVs in the future. Additionally, matching the validity period of functional modification with the integration between BHVs and the host is an overlooked challenge and future direction for BHVs’ crosslinking and modification.
